# The role of Langerhans cells in epidermal homeostasis and pathogenesis of psoriasis

**DOI:** 10.1111/jcmm.15834

**Published:** 2020-09-11

**Authors:** Bei Yan, Nian Liu, Jie Li, Jiaoduan Li, Wu Zhu, Yehong Kuang, Xiang Chen, Cong Peng

**Affiliations:** ^1^ The Department of Dermatology Xiangya Hospital Central South University Changsha China; ^2^ National Clinical Research Center for Geriatric Disorders Xiangya Hospital Changsha China; ^3^ Hunan Key Laboratory of Skin Cancer and Psoriasis Xiangya Hospital Changsha China; ^4^ Hunan Engineering Research Center of Skin Health and Disease Xiangya Hospital Changsha China; ^5^ Xiangya Clinical Research Center for Cancer Immunotherapy Central South University Changsha China

**Keywords:** epidermal homeostasis, inflammatory responses, Langerhans cells, psoriasis

## Abstract

The skin is the main barrier between the human body and the outside world, which not only plays the role of a physical barrier but also functions as the first line of defence of immunology. Langerhans cells (LCs), as dendritic cells (DC) that play an important role in the immune system, are mainly distributed in the epidermis. This review focuses on the role of these epidermal LCs in regulating skin threats (such as microorganisms, ultraviolet radiation and allergens), especially psoriasis. Since human and mouse skin DC subsets share common ontogenetic characteristics, we can further explore the role of LCs in psoriatic inflammation.

## 1 INTRODUCTION OF PSORIASIS

Psoriasis is an inflammatory epidermal proliferative dermatosis which affects 2%‐3% of the world's population.^1^ Clinically, psoriasis is characterized by clear boundaries of skin lesions, erythema and scales on the skin.^2^ Psoriasis was not initially considered as a disease, but a dysfunction of keratinocytes in the epidermis.^3^ A large number of studies have shown that the innate and adaptive immune responses of cells, especially the T‐cell–mediated system, play an important role in the pathogenesis of psoriasis.^4^ In addition, the cytokine network is a key element in psoriasis. The expression levels of interleukin (IL)‐1, tumour necrosis factor (TNF), interleukin‐12 (IL‐12), interleukin‐17 (IL‐17), interleukin‐22 (IL‐22) and interleukin‐23 (IL‐23) in psoriatic skin are significantly increased. Among them, IL‐17A and IL‐22 have the most profound effects on keratinocytes.^5^ In psoriatic lesions, keratinocytes are activated and proliferate much faster than normal keratinocytes.^6^ Not only do cytokines have a strong influence on psoriasis but many immune cells also change greatly in psoriasis. Inflammatory cells in psoriatic skin are mainly composed of dendritic cells (DCs), macrophages, dermal T cells and epidermal neutrophils.^7^ Among these immune cells, dendritic cells increase significantly in psoriatic lesions. Although DCs play a central role in the pathogenesis of psoriasis, the specific role of each DC is not clear. At present, it is believed that psoriasis is caused by the imbalance of the interaction between innate immunity and adaptive immune components of skin cells, and the interconnection between innate immunity and adaptive immune system is realized by cytokines, such as TNF‐α, IFN‐γ and IL‐1.^4^

T cells are considered to be key effector cells that have complex interactive feedback with antigen‐presenting cells, neutrophils, keratinocytes and vascular endothelial cells.^8^ The production of cytokines can be activated by DCs, such as TNF‐α, and after TNF‐α is activated, it can, in turn, activate some secondary mediators and adhesion molecules. These activated mediators and inflammatory factors play an important role in the pathogenesis of psoriasis. DCs are divided into several types that play different roles in the human immune system and perform their respective duties.

## 2 THE ORIGIN OF LANGERHANS CELLS

Dendritic cells play a very important role in the immune system, which are divided into three categories to maintain the balance of the human body: (a) DCs existed in tissue, such as in the interstitial space or the dermis, are called traditional DCs; (b) Plasmacytoid DCs; and (c) Langerhans cells (LCs). LC is a kind of stellate DC located at the base of the epidermis and was discovered by Paul Langerhans in 1868.^9^ One hundred and twenty years later, Nobel laureates Laureate Ralph Steinman and Gerold Schuler found that LCs are derived from dendritic cells that continue to differentiate from lymphoid progenitor cells differentiated from pluripotent haematopoietic stem cells.^10^ However, some recent studies have found that LCs are mainly derived from myeloid progenitor cells.^11^

**FIGURE 1:**
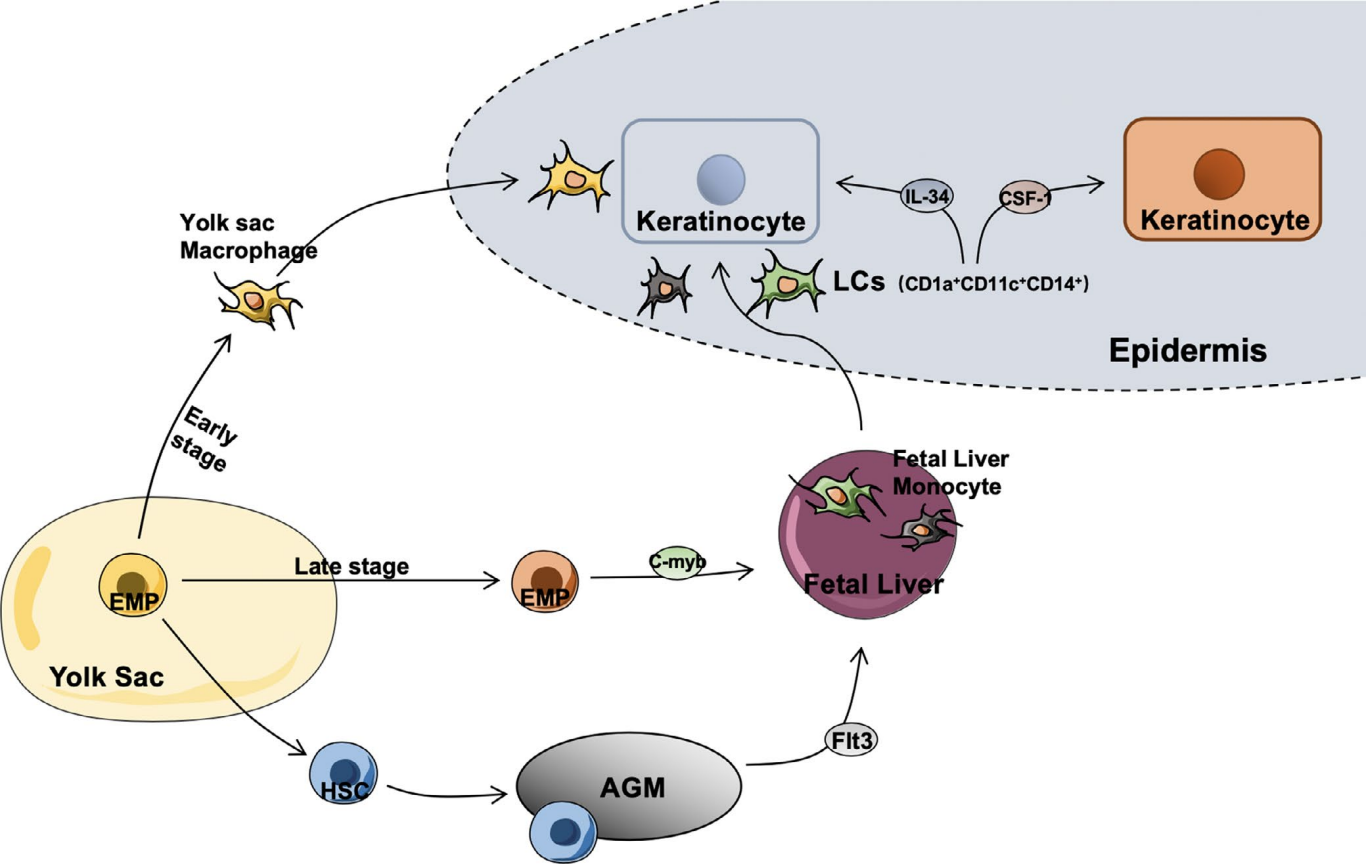
Occurrence and development of Langerhans cells (LCs). LCs are mainly differentiated from primordial erythrocytes‐myeloid progenitors (EMPs) in the yolk sac. There are three pathways to induce and migrate LCs to the epidermis. The development of LCs in the epidermis depends on CSF‐1R, a molecule which could maintain skin homeostasis through combining with IL‐34. LCs could be replaced by mononuclear progenitors, which could bind to CSF‐1, derived from bone marrow during inflammation

It is suggested that LCs were originated from primitive yolk sac haematopoietic cells, which are the precursor cells of yolk sac macrophages (Figure 1). Yolk sac macrophages are produced from the primitive red blood cell‐myeloid progenitor cell (EMP) after approximately 16‐18 days of pregnancy, and some of the LCs migrate to the skin in the form of yolk sac macrophages. Some LCs also migrate to the skin in the form of liver monocytes expressed by c‐myb obtained by late EMP.^12^ The last portion of the pluripotent haematopoietic stem cells (HSCs) produced by LCs through EMP will appear in the aorta‐gonad‐mesonephros (AGM) region of the human body since the 32nd day of pregnancy, when it binds to flt3 and begin to migrate to the foetal liver and then to the bone marrow, enter the skin, where they exist for a long time.^13^ C‐myb and flt3 are necessary kinases in the process of HSC formation.^14^ Therefore, LCs are considered to be a macrophage that retains the function of DCs.^15^ It is mainly located in the epithelial cells of many organs and is first present in the epidermis of the skin.^16^ LCs account for approximately 2%‐3% of skin cells.^17^ The development of LCs migrate to the epidermis depends on CSF‐1R.^18^ This is also different from most DC subsets, which depend on another receptor, the tyrosine kinase FLT3.^19^ There are two kinds of ligands for CSf‐1R. (a) IL‐34, derived from keratinocytes, and (b) CSF1, derived from neutrophils, have different ligands in different skin states. CSF1R mainly binds to IL‐34 in normal skin.^20^ During inflammation, it will be replaced by bone marrow‐derived mononuclear progenitor cells and bind to CSF‐1.^21^ IL‐34 is produced in the epidermis of embryonic developmental skin. As a ligand of CSf‐1R, it plays an important role in the development of LCs and the maintenance of balance in vivo.^22^, ^23^

Langerhans cells are epidermal cells characterized by the expression of Langerin (CD207) and CD1a. LCs play a clear role of antigen‐presenting cells in skin inflammation.^24^ Langerin is a type II type c lectin receptor, called Birbeck granule, which is involved in the presentation of non‐peptide antigens to T cells^25^ and is expressed only in LCs in the skin.^26^ When confronted with pathogens or allergens, LCs act as antigen presentation function triggering a series of immune responses by migrating from the epidermis to the lymph nodes by presenting antigens to T‐regulatory cells. The migration of LCs in the epidermis during homeostasis and inflammation comes from different progenitor cells, which perform their respective functions.

## 3 MIGRATION OF LANGERHANS CELLS DURING HOMEOSTASIS AND INFLAMMATION

Although LCs in the epidermis are mainly derived from myeloid progenitor cells, they can exist for a long time after migration to the epidermis and can renew themselves in a steady state.^27^ After the mature LCs migrate to the epidermis, the hydrophilic E‐cadherin binds to the surrounding keratinocytes, which can maintain the stability of LCs in the epidermis.^28^ E‐cadherin is a transmembrane glycoprotein that mediates Ca2^+^‐dependent intercellular adhesion. It is the main component of adhesion junction and helps to maintain the integrity of the epidermal barrier.^29^ LCs without E‐cadherin showed significant morphological change: more round cell bodies and fewer dendrites. However, the absence of E‐cadherin did not affect the turnover, maturation, migration and function of LCs, and E‐cadherin was down‐regulated during LCs mobilization and migration from the epidermis.^28^ The non‐haematopoietic origin of TGF‐β1 is considered to play an important role in promoting LC renewal.^30^ In vivo, TGF‐β1 is secreted by white blood cells and non‐haematopoietic cells (including keratinocytes). It has multiple effects in the immune system.^31^ There are three isotypes of TGF‐β, among which TGF‐β1 is the main isotype in the immune system. The development of LCs does not require haematopoietic‐derived TGF‐β1. TGFβ‐1 from LCs directly acts on LCs through the autocrine/paracrine loop, which is necessary for LC genesis and survival,^32^ and it is consistent with the ability of keratinocytes to produce M‐CSF and TGF‐β1.^33^ The microenvironment of LCs (for example, keratinocyte signal) can induce a large number of resident langerin^+^ LCs to be proliferated^20^; therefore, it is a crucial element of controlling LC homeostasis.

Exposure to UVB radiation or irritants induces the migration of LCs which originally resident in the epidermis and promotes the migration of bone marrow‐derived Gr‐1^hi^ mononuclear cells to the epidermis.^20^ Among them, CCCTC binding factor (CTCF) can promote the outflow of LCs from the epidermis.^34^ CTCF plays a variety of roles in the haematopoietic lineage, which can regulate the early development of thymocytes and participate in the development and the operation of macrophages.^35^, ^36^ Gr‐1 (also known as Ly‐6c/G) is a monocyte with high expression of monocyte markers, and it is the precursor of LC in inflammatory epidermis.^18^ Macrophage inflammatory protein (MIP)‐3 α, as a chemokine produced by epidermis, plays a central role in the summons of LC Precursors to epidermis.^37^ Gr‐1^hi^ monocytes express the inflammatory chemokine CCR2 receptor, which in turn promotes the secretion of proinflammatory CCR2 (monocyte chemoattractant protein 1), CCR7 and other chemokines in the skin.^20^, ^38^

## 4 THE FUNCTION OF LANGERHANS CELLS IN THE INFLAMMATORY RESPONSE

Previous studies have told us that B cells and CD8^+^T cells are the main effectors of the adaptive immune system, while CD4^+^T cells can regulate the function of other types of lymphocytes.^39^ The CD4^+^T‐cell compartment is particularly complex because it includes Th1, Th2, Th17 and T follicle helper (TFH) cells.^40^, ^41^ Among them, TFH cells are very important for the establishment of the germinal centre (GC), and they provide help for the expansion, selection and affinity maturation of antigen‐specific B cells.^42^ DCs and LCs, as antigen‐presenting cells (APCs), present endogenous peptides under the background of major histocompatibility complex (MHC) I molecules and exogenous peptides under the background of MHC II molecules to CD8^+^ and CD4^+^T cells, respectively,^43^, ^44^ lead to induction of the adaptive immune response and gain the ability to capture and present exogenous antigens through MHC I molecules.^45^ However, the secretion of IL‐12 cytokines mediated by DCs and LCs can stimulate the activation of NK and γδT cells, thus destroy the target cells of MHC I molecules which lack of self‐recognition. Meanwhile, NK and γδT can provide positive feedback for the maturation of DCs and LCs and promote the transmission of innate and adaptive immune responses.^46^

**FIGURE 2:**
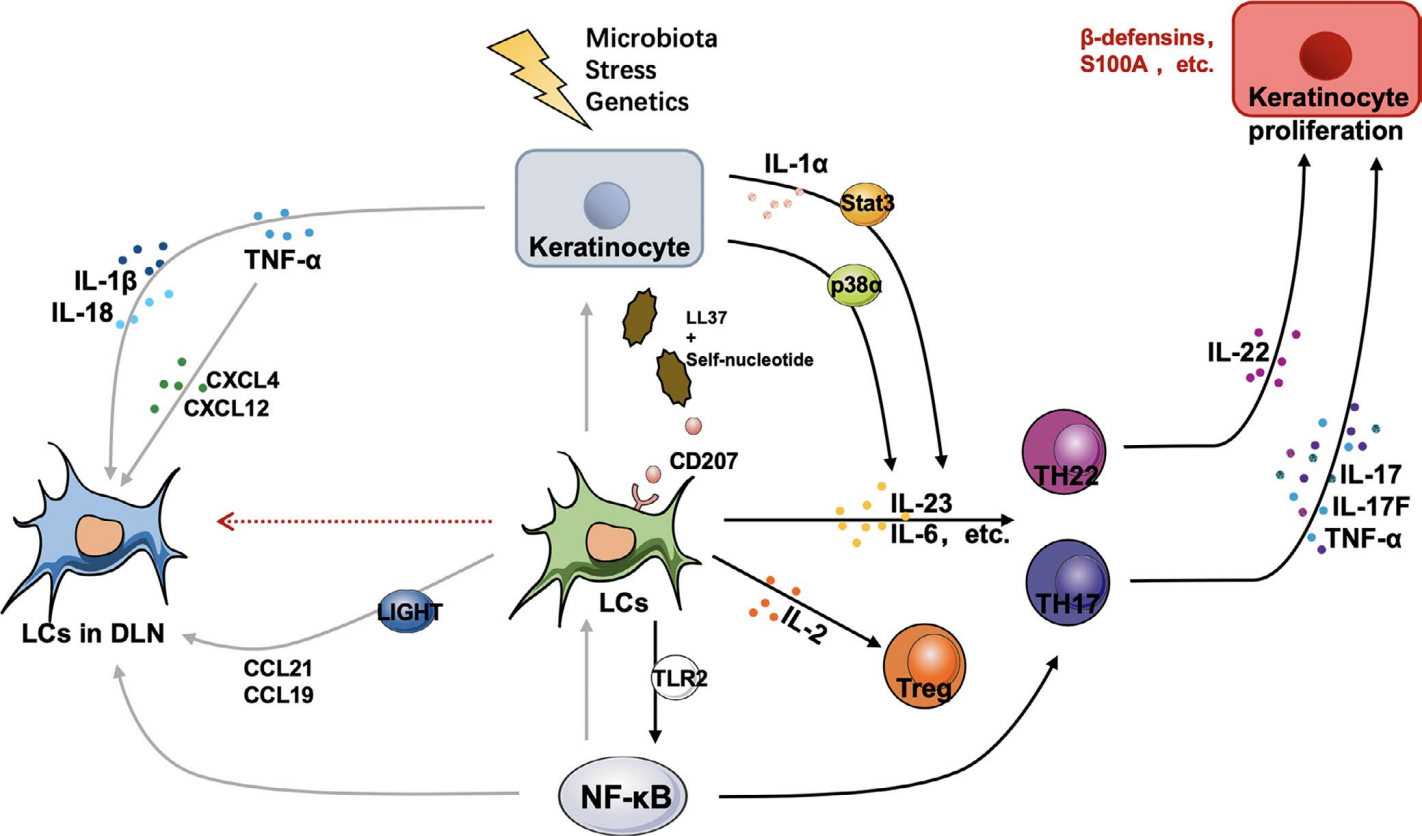
The role of Langerhans cells (LCs) in the occurrence and development of psoriasis. KCs stimulated by stress stimulation (such as cytokines) can stimulate the self‐nucleotide and antimicrobial peptide LL‐37 complex to activate epidermal LCs, inducing keratinocytes to specifically produce a variety of cytokines to promote the migration of LCs to lymph nodes (DLNs) and T‐cell activation. LCs can also activate the LIGHT‐LT β R signal axis to activate the expression of CCL21/CCL19 in DLN. At the same time, epidermal LCs can produce IL‐2, IL‐23 and TLR2. Among them, IL‐2 can promote the proliferation of Treg cells; TLR2 signal mediates the activation of the transcription factor NF‐κB, promotes LC maturation and migration to lymph nodes and induces Th17 differentiation. The cytokines produced by LCs promote Th17 and Th22 to activate the expression of IL‐17, IL‐22 and TNF‐α and promote the proliferation of keratinocytes and other features of psoriasis. Proliferated KCs once again release cytokines, antimicrobial peptides and chemokines to infiltrate immune cells, further enhancing the role of activated T cells and forming a positive feedback loop between psoriatic epidermal cells and the immune system

Antigen‐presenting cells in the epidermis mainly consist of LCs, and their marker langerin mediates recognition by interacting with sugar conjugates such as mannan, which has a high mannose structure, or β‐glucan, and expressed on the surface of pathogens.^43^, ^47^ Langerin mediates ligand internalization for antigen processing and presentation; thus, they can be used to specifically transport antigens that bind to polysaccharides or α‐langerin antibodies to LCs (Figure 2).^43^ The activation of LCs after the binding of langerin to epidermal antigen can directly promote the IL‐2‐mediated signal conditions to induce the proliferation of Treg cells.^48^ The migration of LCs from the epidermis is controlled by IL‐1β, IL‐18 and tumour necrosis factor‐α (TNF‐α), which are specifically produced by keratinocytes.^49^ At the same time, the expression of TNF‐α induces the expression of CXCL12 in skin fibroblasts.^50^ CXCR4 has also been shown to play a key role in the maturation and lymph node migration of DC.^51^

Therefore, the CXCR4‐CXCL12 axis controls the migration of LCs to lymph nodes (DLNs). Other studies have shown that LCs can activate the LIGHT‐LTβR signal axis as well as the CCL21/CCL19 expression in lymphatic endothelial cells, thus promote LCs' migration.^52^

At this time, LCs preferentially induce the proliferation of CD4^+^T cells, which differentiate and secrete Th1 and Th2 cytokines, and the initiation and cross‐activation of immature CD8^+^T cells become apparent.^39^, ^53^ LCs are also involved in the differentiation of TFH cells, which is also very important for the induction of TFH cells and the production of germinal centres.^54^ At the same time, LCs derived from CD34^+^ progenitor cells cultured in vitro can effectively promote the proliferation of CD8^+^T cells after internalization of soluble peptides.^39^ LCs are also equipped with membrane‐spanning Toll‐like receptors (TLRs) to recognize pathogen‐related molecular patterns (PAMPs).^55^ TLR2 signalling is mediated by MyD88‐dependent Toll‐mediated pathways, including phosphorylation of IL‐1R‐associated kinase (IRAK), which eventually leads to the activation of transcription factors such as NF‐κB.^56^ These processes promote the maturation of LCs and increase the expression of costimulatory molecules and MHC II, thus enhancing the ability antigen presentation ability of LCs. The cells also participate in the synergistic release of proinflammatory chemokines, including T‐cell–stimulating factor and IL‐6, IL‐18 and TNF‐α, the expression of adhesion molecules and the interaction between immature T cells and cells migrate to lymph nodes, inducing the differentiation of Th17.^57^, ^58^ At the same time, after the skin is triggered by microbial‐derived ligands (such as lipopolysaccharide [LPS] or bacterial cell wall compound peptidoglycan [PGN]) or stimulated by toxins or stimulants (risk‐related molecular model, DAMP),^59^ the expression levels of the LCs maturation markers CD40, CD80, HLA‐DR and CCR7 and the release of IL‐1β and IL‐23 are greatly enhanced. These factors are potential Th17‐driven cytokines.^58^ In the epidermis, TLR‐2 detects the composition of mycobacteria and Gram‐positive bacteria with its related receptors TLR‐1 and TLR‐6. Exposure to bacterial antigens also leads to the phosphorylation of the MAP kinase ERK, which promotes LCs maturation, which may contribute to the tolerance of LCs.^55^ To further study LCs, scientists have identified LC‐like cells that can be cultured in vitro.

## 5 DIFFERENCES BETWEEN LCS AND LC‐LIKE CELLS IN EPIDERMIS

According to the expression of CD11c and CD1a, the first two species of DCs in human peripheral blood were identified as CD11c^+^DC, composed of the main CD1a^+^/CD11c^+^ component (group 1) and a small amount of a CD1a^−^/CD11c^+^ component (group 2). The CD11c components are monocyte‐like and have GM‐CSF receptors; the third species is composed of CD1a^−^/CD11c^−^ dendritic cells (group 3), which are similar to plasma cell‐like T cells and do not express GM‐CSF receptors. Among them, CD1a^+^/CD11c^+^/CD14^−^DC is considered to be the direct precursor of LCs.^60^ CD34 peripheral blood monocytes cultured in GM‐CSF, IL‐4 and TNF‐α differentiate into mature DCs.^61^, ^62^, ^63^ If TGF‐β1 is added to the culture medium, LCs may be formed.^64^ As a substitute for human skin, many functional studies of LCs use the above‐mentioned LCs derived from CD34^+^ haematopoietic progenitor cells and compare epidermal LCs and LC‐like cells.

Thymic interstitial lymphopoietin (TSLP) is an interleukin‐(IL)‐7‐like cytokine. Human LCs treated with TSLP experience phenotypic and functional maturation.^65^ Epidermal LCs treated with TSLP can induce CD4 helper T cells to produce inflammatory TH2 cytokines and thymus and activation‐regulated chemokine (TARC)/CCL17.^66^, ^67^ Unlike epidermal LCs, TSLP stimulation does not enhance the survival and maturation of LC‐like cells derived from CD34^+^. In addition, TSLP does not increase the immunostimulatory ability of these cells, does not induce them to produce TARC/CCL17 and cannot promote the expression of inflammatory Th2 cytokines in CD4^+^helper T cells.^63^ Therefore, LC‐like cells cannot completely replace epidermal LCs in research. However, there is also a view states that TSLP does not play a major role in controlling the proliferation of LCs in the process of development and inflammation.

## 6 THE ROLE OF LANGERHANS CELLS IN PSORIASIS

### 6.1 Patients with psoriasis

Since 1970, research data on the number of LCs in psoriasis have been debated, and different reports have detected an increase,^68^, ^69^ decrease^70^, ^71^ or stability^72^, ^73^ of LCs in the epidermis of patients with psoriasis. However, there is a great difference in the number of LCs between normal subjects and patients with psoriasis, and the results of different experiments are different, which may related to the redistribution of LCs and the common surfacers of inflammatory DCs.^74^ The onset of psoriasis has two different age groups. Early onset occurs before the age of 40, accounting for 75% of cases, while late onset occurs after the age of 40, with a peak between 55 and 60 years old. Although these two types of psoriasis look very similar clinically, their pathogenic genes are different. The HLA‐CW6 allele is associated with early‐onset psoriasis but has little association with late‐onset psoriasis.^75^ The mobilization and migration of LCs in the epidermis of patients with early‐onset psoriasis were seriously impaired, and the impaired migration is closely related to bone marrow‐derived LCs.^76^ Among them, early‐onset chronic plaque psoriasis (CPP) is strongly linked to the inhibition of LCs migration in epidermal. LCs migration damage can be detected in both guttate psoriasis and CPP lesions. LCs migration returned to normal after remission of guttate psoriasis, but LCs will be severely damaged and unable to recover if migrated by guttate psoriasis progressed to CPP.^77^, ^78^ The significant decrease or completely absent of LC migration in early‐onset psoriasis is mainly caused by IL‐1β, TNF‐α and contact allergen.^77^ In late‐onset psoriasis, keratinocytes do not secrete cytokines that inhibit LC migration.^79^ This may due to the fact that LCs respond to IL‐1β rather than TNF‐α. The failure of exogenous TNF‐α to mobilize LCs does not necessarily reflect the non‐response to TNF‐α. Since LC migration needs to receive signals from both TNF‐α and IL‐1β, unresponsiveness in this case may be a secondary factor in the production of IL‐1β, biological activation and/ or impaired signal transduction.^80^

It was reported that LCs induce the proliferation of CD4^+^T cells under inflammatory conditions, while CD4^+^T cells, CD8^+^T cells and γδT cells produce IL‐17, IL‐17A, IL‐17F, IL‐21 and IL‐22 as cytokines of Th17 cells.^81^ As described in Figure 2, the increased activity of p38α mitogen‐activated protein kinase is related to human susceptibility to psoriasis.^82^ The activity of p38 was also increased in KCs stimulated by stress stimulation (such as cytokines and ultraviolet radiation).^83^ The p38α signal in LCs rather than DCs specifically promotes the production of IL‐17 in γδT and CD4^+^T cells by secreting IL‐23 and IL‐6, which are essential for the development of the psoriasis.^84^

In addition, studies have shown that the activation of Stat3 in keratinocytes may affect the activation of LCs at least partly through IL‐1 α stimulation, and their existence is related to the occurrence or aggravation of psoriasis, called the Koebner phenomenon, which is caused by IL‐23.^85^, ^86^ This activation leads to the intraepidermal circuit of KC‐LCs and the activation of the IL‐23/IL‐17 axis, which leads to the occurrence and development of psoriasis. Human β‐defensin 3 (HBD3) is a small antimicrobial peptide that has a chemotactic effect on immune cells and plays a small role in promoting the development of psoriasis by inducing the increase of IL‐23 produced by epidermal LCs.^87^, ^88^

Moreover, LCs can induce whole peripheral T cells and immature CD4^+^T cells to produce IL‐22, a cytokine that mainly acts on epithelial cells. In the skin, it produces antimicrobial proteins, such as HBD3, indicating that this cytokine is involved in the defence of early hosts against microbial pathogens,^89^ and mediates keratinocyte proliferation and epidermal hyperplasia, which is thought to play an important role in inflammatory diseases with obvious epidermal acanthosis, such as psoriasis.^89^ It has been shown that not only Th17 cells but also T helper cells can produce IL‐22, and the type of T helper cells that only produce IL‐22 is called Th22.^90^ It is considered that the DCs in the skin, especially the IL‐22 cytokines induced by LCs to penetrate into the skin, are produced by Th22 cells.^91^ Because there are still many differences between LCs and human epidermal LCs in experiments performed in vitro, more people choose to create mouse models to study LCs.

### 6.2 Mouse model of psoriasis

Human skin is different from mouse skin in terms of its anatomy. Human skin is divided into epidermis and dermis, while the epidermis is divided into four layers: a stratum corneum, a transparent layer, a granular layer and a germinal layer, all composed of lamellar flat epithelium. The dermis is divided into two layers: a papillary layer and a reticular layer, and mainly composed of dense connective tissue. The mouse skin has only 2‐3 layers, covered under dense hair follicles.^92^ The DCs of mouse skin can be divided into LCs, Langerin^+^DCs and Langerin‐DCs. Dermal DCs consist of two main subgroups: Langerin‐DC (CD103^+^CD11b^−^) and Langerin‐DC (CD103^−^CD11b^+^). The difference between LCs and dermal Langerin‐DCs lies in the high expression of CD11b and epithelial cell adhesion molecule (EpCAM) as well as the low expression of CD103.^93^ At present, the study of LCs mainly uses langerin‐diphtheria toxin A (DTA) or diphtheria toxin receptor (DTR) mice with LC gene defects to establish the model of psoriatic dermatitis. In addition, three independent mouse lines have been designed to effectively ablate the endogenous Langerin site introduced by LCs; thus, the primate diphtheria toxin receptor (DTR) and the Langerin‐DTR (muLangerin‐DTR) mouse strain were established^94^ using human genomic BAC DNA, human Langerin‐DTA (huLangerin‐DTA) and huLangerin‐DTR transgenic mice containing Langerin loci expressing active Diphtheria toxin or DTR.^95^

**FIGURE 3:**
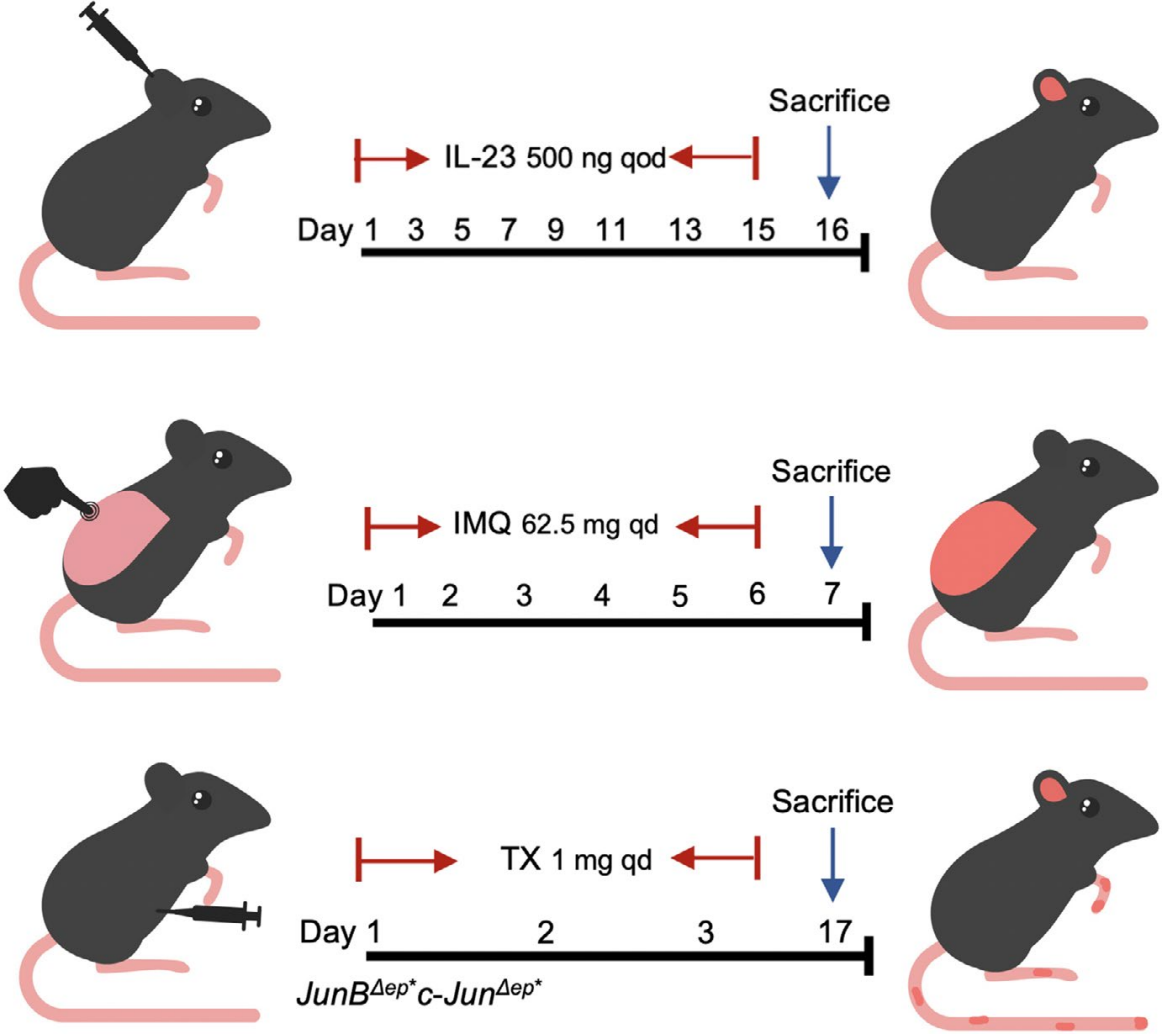
The three kinds of way to establish Psoriasis‐like Mouse Model. The first type of psoriasis‐like mouse model was intradermal injection of 500 mg of IL‐23 every other day for 15 days; the second kind was to smear 62.5 mg of imiquimod on the back every day for 6 days; and the third kind was intraperitoneal injection of tamoxifen for three consecutive days of 1 mg in DKO* mice, which could lead to acanthosis, incomplete keratosis and mixed inflammatory infiltration after 14 days

The mouse psoriatic dermatitis models have been generated by different ways, such as local administration of IL‐23, topical application Imiquimod (IMQ) as ligands of TLR7 and TLR8, and the deletion of cre recombinant enzyme in keratin 5 expressing cells (Jun^f/f^ JunB^f/f^ K5creER = DKO * mice) induced by tamoxifen (TX) lead to acanthosis, hypokeratosis and mixed inflammatory infiltration in mice with common psoriasis^96^, ^97^, ^98^ (Figure 3). The severity of dermatitis induced by IMQ in LC gene‐deficient mice is significantly lower than that in wild‐type mice. It is considered that LCs play a very important role in the inflammatory response of psoriatic dermatitis induced by IMQ.^99^ The development of these three common psoriatic dermatitis models is very dependent on IL‐23/IL‐17/IL‐22 axis.^96^, ^100^, ^101^

In the comparison of the skin transcriptional group of the psoriasis mouse model, the transcriptional group in the IL‐23 injection model was most similar to the gene expression pattern found in psoriatic skin.^102^ Subcutaneous injection of IL‐23, together with IL‐12 and TNF produced by LCs and DCs, activates αβT and γδT cells to produce IL‐22 and IL‐17A/F, which stimulate keratinocyte proliferation, release S100 protein and β‐defensin, and promote the production of growth factors and chemokines in psoriasis.^100^

The accumulation of LCs in the epidermis of psoriatic skin lesions induced by IMQ is mainly related to the local proliferation of the LC pool. Local treatment with IMQ for 5‐7 days can induce the increase of LC, the production of IL‐23, and γδT cells which produce IL‐17A in mice.^103^ These γδT cells can migrate to the epidermis of IMQ‐treated skin through CCR6 to further develop psoriasis dermatoses.^104^ CCR6 was demonstrated to be expressed on all peripheral T cells expressing IL‐17A/F and IL‐22.^105^ Other studies have shown that Langerin‐DTR mice could reduce the inflammation of psoriasis‐like dermatitis induced by IMQ and reduce the number of γδT cells produced by permeable IL‐17A. In the mouse model, it was found that CD1a, a lipid‐presenting molecule heavily expressed on LCs, could amplify the inflammatory response mediated by Th17 cells in response to self‐lipid antigens.^106^ The role of Langerin epidermal LC in inducing the TFH‐cell response was confirmed in huLangerin‐DTR mice.^54^ The mouse model of psoriasis induced by IMQ mentioned above is only used to study the early stages of the disease.^107^ Some studies have adjusted the IMQ treatment time to 14 days to better simulate human conditions and study the late stages of the disease. Langerin‐DTR mice can induce IMQ‐induced psoriatic dermatitis and promote a large number of epidermal neutrophils in the late stage.^108^ However, it is still controversial whether this method can really represent the late stage of psoriasis.

Interestingly, the third type of psoriatic model using DKO* mice showed chronic inflammation, remained stable over a long period of time and was thought to mimic the chronic phase of human disease. Studies have shown that in this mouse model, bone marrow‐derived LCs decreased significantly, while IL‐23 content increased, but did not affect the expression levels of IL‐17 and IL‐22.^109^ The chronic development of this psoriatic dermatitis model may depend on the direct action of IL‐23 on keratinocytes.^110^ Therefore, the proliferative and activated LCs found in the epidermis In melanoma, the decrease in the number of LC is due to the loss of the antigen presentation function of LC, which promotes the continued survival of tumour cells' immunomodulatory effect in the late stage.

## 7 CONCLUSION

As immune cells of the epidermis, LC is essential for sensing danger and triggering congenital and adaptive protective responses in the body. In other skin diseases, such as Atopic dermatitis, LC depletion can help to relief the disease.^111^ In melanoma, the decrease in the number of LC is due to the loss of the antigen presentation function of LC, which promotes the continued survival of tumour cells.^112^ However, the role and function in psoriasis are controversial; it is possible that there is a certain heterogeneity of LC, there are new functional subsets have not been found, which is also an important content of future research on the role of LC in psoriasis.

## CONFLICT OF INTEREST

The authors declare that they have no conflict of interest.

## AUTHOR CONTRIBUTION

**Bei Yan:** Writing – original draft (equal). **Nian Liu:** Methodology (equal). **Jie Li:** Writing – review and editing (equal). **Jiaoduan Li:** Methodology (equal). **Wu Zhu:** Formal analysis (equal). **Yehong Kuang:** Formal analysis (equal). **Xiang Chen:** Conceptualization (equal); project administration (equal). **Cong Peng:** Writing – review and editing (equal).

## References

[1] Dubois Declercq S , Pouliot R . Promising new treatments for psoriasis. Sci World J. 2013;2013:980419. 10.1155/2013/980419 PMC3713318 23935446

[2] Griffiths CE , Barker JN . Pathogenesis and clinical features of psoriasis. Lancet. 2007;370(9583):263‐271. 17658397 10.1016/S0140-6736(07)61128-3

[3] Killeen ME , Ferris L , Kupetsky EA , Falo L Jr , Mathers AR . Signaling through purinergic receptors for ATP induces human cutaneous innate and adaptive Th17 responses: implications in the pathogenesis of psoriasis. J Immunol. 2013;190(8):4324‐4336. 23479230 10.4049/jimmunol.1202045 PMC3622186

[4] Mekori YA , Hershko AY , Frossi B , Mion F , Pucillo CE . Integrating innate and adaptive immune cells: mast cells as crossroads between regulatory and effector B and T cells. Eur J Pharmacol. 2016;778:84‐89. 25941086 10.1016/j.ejphar.2015.03.087

[5] Nograles KE , Zaba LC , Guttman‐Yassky E , et al. Th17 cytokines interleukin (IL)‐17 and IL‐22 modulate distinct inflammatory and keratinocyte‐response pathways. Br J Dermatol. 2008;159(5):1092‐1102. 18684158 10.1111/j.1365-2133.2008.08769.x PMC2724264

[6] Iizuka H . Epidermal turnover time. J Dermatol Sci. 1994;8(3):215‐217. 7865480 10.1016/0923-1811(94)90057-4

[7] Nestle FO , Conrad C . The IL‐12 family member p40 chain as a master switch and novel therapeutic target in psoriasis. J Invest Dermatol. 2004;123(6):xiv‐xv. 10.1111/j.0022-202X.2004.23488.x 15610500

[8] Dombrowski Y , Schauber J . Cathelicidin LL‐37: a defense molecule with a potential role in psoriasis pathogenesis. Exp Dermatol. 2012;21(5):327‐330. 22509827 10.1111/j.1600-0625.2012.01459.x

[9] De Panfilis G . Paul Langerhans' death centennial, July 20, 1988. J Invest Dermatol. 1988;91(3):283. 3045211 10.1111/1523-1747.ep12470473

[10] Schuler G , Steinman RM . Murine epidermal Langerhans cells mature into potent immunostimulatory dendritic cells in vitro. J Exp Med. 1985;161(3):526‐546. 3871837 10.1084/jem.161.3.526 PMC2187584

[11] Katz SI , Tamaki K , Sachs DH . Epidermal Langerhans cells are derived from cells originating in bone marrow. Nature. 1979;282(5736):324‐326. 503208 10.1038/282324a0

[12] Hoeffel G , Chen J , Lavin Y , et al. C‐Myb(+) erythro‐myeloid progenitor‐derived fetal monocytes give rise to adult tissue‐resident macrophages. Immunity. 2015;42(4):665‐678. 25902481 10.1016/j.immuni.2015.03.011 PMC4545768

[13] Collin M , Milne P . Langerhans cell origin and regulation. Curr Opin Hematol. 2016;23(1):28‐35. 26554892 10.1097/MOH.0000000000000202 PMC4685746

[14] Schulz C , Perdiguero EG , Chorro L , et al. A lineage of myeloid cells independent of Myb and hematopoietic stem cells. Science. 2012;336(6077):86‐90. 22442384 10.1126/science.1219179

[15] Guilliams M , Ginhoux F , Jakubzick C , et al. Dendritic cells, monocytes and macrophages: a unified nomenclature based on ontogeny. Nat Rev Immunol. 2014;14(8):571‐578. 25033907 10.1038/nri3712 PMC4638219

[16] Romani N , Clausen BE , Stoitzner P . Langerhans cells and more: langerin‐expressing dendritic cell subsets in the skin. Immunol Rev. 2010;234(1):120‐141. 20193016 10.1111/j.0105-2896.2009.00886.x PMC2907488

[17] Romani N , Holzmann S , Tripp CH , Koch F , Stoitzner P . Langerhans cells ‐ dendritic cells of the epidermis. APMIS. 2003;111(7–8):725‐740. 12974775 10.1034/j.1600-0463.2003.11107805.x

[18] Ginhoux F , Tacke F , Angeli V , et al. Langerhans cells arise from monocytes in vivo. Nat Immunol. 2006;7(3):265‐273. 16444257 10.1038/ni1307 PMC4727824

[19] Mende I , Karsunky H , Weissman IL , Engleman EG , Merad M . Flk2+ myeloid progenitors are the main source of Langerhans cells. Blood. 2006;107(4):1383‐1390. 16263793 10.1182/blood-2005-05-1878 PMC1895406

[20] Merad M , Manz MG , Karsunky H , et al. Langerhans cells renew in the skin throughout life under steady‐state conditions. Nat Immunol. 2002;3(12):1135‐1141. 12415265 10.1038/ni852 PMC4727838

[21] Wang Y , Szretter KJ , Vermi W , et al. IL‐34 is a tissue‐restricted ligand of CSF1R required for the development of Langerhans cells and microglia. Nat Immunol. 2012;13(8):753‐760. 22729249 10.1038/ni.2360 PMC3941469

[22] Wang Y , Colonna M . Interkeukin‐34, a cytokine crucial for the differentiation and maintenance of tissue resident macrophages and Langerhans cells. Eur J Immunol. 2014;44(6):1575‐1581. 24737461 10.1002/eji.201344365 PMC4137395

[23] Wang Y , Bugatti M , Ulland TK , Vermi W , Gilfillan S , Colonna M . Nonredundant roles of keratinocyte‐derived IL‐34 and neutrophil‐derived CSF1 in Langerhans cell renewal in the steady state and during inflammation. Eur J Immunol. 2016;46(3):552‐559. 26634935 10.1002/eji.201545917 PMC5658206

[24] Gomez Perdiguero E , Klapproth K , Schulz C , et al. Tissue‐resident macrophages originate from yolk‐sac‐derived erythro‐myeloid progenitors. Nature. 2015;518(7540):547‐551. 25470051 10.1038/nature13989 PMC5997177

[25] Hunger RE , Sieling PA , Ochoa MT , et al. Langerhans cells utilize CD1a and langerin to efficiently present nonpeptide antigens to T cells. J Clin Invest. 2004;113(5):701‐708. 14991068 10.1172/JCI19655 PMC351318

[26] Valladeau J , Ravel O , Dezutter‐Dambuyant C , et al. Langerin, a novel C‐type lectin specific to Langerhans cells, is an endocytic receptor that induces the formation of Birbeck granules. Immunity. 2000;12(1):71‐81. 10661407 10.1016/s1074-7613(00)80160-0

[27] Chorro L , Sarde A , Li M , et al. Langerhans cell (LC) proliferation mediates neonatal development, homeostasis, and inflammation‐associated expansion of the epidermal LC network. J Exp Med. 2009;206(13):3089‐3100. 19995948 10.1084/jem.20091586 PMC2806478

[28] Brand A , Diener N , Zahner SP , et al. E‐cadherin is dispensable to maintain Langerhans cells in the epidermis. J Invest Dermatol. 2020;140(1):132‐142.e133. 31260672 10.1016/j.jid.2019.06.132

[29] Van den Bossche J , Van Ginderachter JA . E‐cadherin: from epithelial glue to immunological regulator. Eur J Immunol. 2013;43(1):34‐37. 23229729 10.1002/eji.201243168

[30] Borkowski TA , Letterio JJ , Mackall CL , et al. A role for TGFbeta1 in langerhans cell biology. Further characterization of the epidermal Langerhans cell defect in TGFbeta1 null mice. J Clin Invest. 1997;100(3):575‐581. 9239404 10.1172/JCI119567 PMC508224

[31] Li MO , Wan YY , Sanjabi S , Robertson AK , Flavell RA . Transforming growth factor‐beta regulation of immune responses. Annu Rev Immunol. 2006;24:99‐146. 16551245 10.1146/annurev.immunol.24.021605.090737

[32] Kaplan DH , Li MO , Jenison MC , Shlomchik WD , Flavell RA , Shlomchik MJ . Autocrine/paracrine TGFbeta1 is required for the development of epidermal Langerhans cells. J Exp Med. 2007;204(11):2545‐2552. 17938236 10.1084/jem.20071401 PMC2118472

[33] Chodakewitz JA , Lacy J , Edwards SE , Birchall N , Coleman DL . Macrophage colony‐stimulating factor production by murine and human keratinocytes. Enhancement by bacterial lipopolysaccharide. J Immunol. 1990;144(6):2190‐2196. 2179407

[34] Kim T‐G , Kim M , Lee J‐J , et al. CCCTC‐binding factor controls the homeostatic maintenance and migration of Langerhans cells. J Allergy Clin Immunol. 2015;136(3):713‐724. 25936568 10.1016/j.jaci.2015.03.033

[35] Heath H , Ribeiro de Almeida C , Sleutels F , et al. CTCF regulates cell cycle progression of alphabeta T cells in the thymus. EMBO J. 2008;27(21):2839‐2850. 18923423 10.1038/emboj.2008.214 PMC2580790

[36] Nikolic T , Movita D , Lambers MEH , et al. The DNA‐binding factor Ctcf critically controls gene expression in macrophages. Cell Mol Immunol. 2014;11(1):58‐70. 24013844 10.1038/cmi.2013.41 PMC4002140

[37] Dieu‐Nosjean MC , Massacrier C , Homey B , et al. Macrophage inflammatory protein 3alpha is expressed at inflamed epithelial surfaces and is the most potent chemokine known in attracting Langerhans cell precursors. J Exp Med. 2000;192(5):705‐718. 10974036 10.1084/jem.192.5.705 PMC2193271

[38] Merad M , Hoffmann P , Ranheim E , et al. Depletion of host Langerhans cells before transplantation of donor alloreactive T cells prevents skin graft‐versus‐host disease. Nat Med. 2004;10(5):510‐517. 15098028 10.1038/nm1038 PMC4727841

[39] Klechevsky E , Morita R , Liu M , et al. Functional specializations of human epidermal Langerhans cells and CD14+ dermal dendritic cells. Immunity. 2008;29(3):497‐510. 18789730 10.1016/j.immuni.2008.07.013 PMC2688399

[40] Weaver CT , Hatton RD , Mangan PR , Harrington LE . IL‐17 family cytokines and the expanding diversity of effector T cell lineages. Annu Rev Immunol. 2007;25:821‐852. 17201677 10.1146/annurev.immunol.25.022106.141557

[41] King C , Tangye SG , Mackay CR . T follicular helper (TFH) cells in normal and dysregulated immune responses. Annu Rev Immunol. 2008;26:741‐766. 18173374 10.1146/annurev.immunol.26.021607.090344

[42] Crotty S . Follicular helper CD4 T cells (TFH). Annu Rev Immunol. 2011;29:621‐663. 21314428 10.1146/annurev-immunol-031210-101400

[43] Fehres CM , Duinkerken S , Bruijns SCM , et al. Langerin‐mediated internalization of a modified peptide routes antigens to early endosomes and enhances cross‐presentation by human Langerhans cells. Cell Mol Immunol. 2017;14(4):360‐370. 26456691 10.1038/cmi.2015.87 PMC5380941

[44] Lambrecht BN , Salomon B , Klatzmann D , Pauwels RA . Dendritic cells are required for the development of chronic eosinophilic airway inflammation in response to inhaled antigen in sensitized mice. J Immunol. 1998;160(8):4090‐4097. 9558120

[45] Heath WR , Belz GT , Behrens GMN , et al. Cross‐presentation, dendritic cell subsets, and the generation of immunity to cellular antigens. Immunol Rev. 2004;199:9‐26. 15233723 10.1111/j.0105-2896.2004.00142.x

[46] Merad M , Fong L , Bogenberger J , Engleman EG . Differentiation of myeloid dendritic cells into CD8alpha‐positive dendritic cells in vivo. Blood. 2000;96(5):1865‐1872. 10961888

[47] Feinberg H , Taylor ME , Razi N , et al. Structural basis for langerin recognition of diverse pathogen and mammalian glycans through a single binding site. J Mol Biol. 2011;405(4):1027‐1039. 21112338 10.1016/j.jmb.2010.11.039 PMC3065333

[48] Kitashima DY , Kobayashi T , Woodring T , et al. Langerhans cells prevent autoimmunity via expansion of keratinocyte antigen‐specific regulatory T cells. EBioMedicine. 2018;27:293‐303. 29307572 10.1016/j.ebiom.2017.12.022 PMC5828466

[49] Cumberbatch M , Dearman RJ , Kimber I . Langerhans cells require signals from both tumour necrosis factor‐alpha and interleukin‐1 beta for migration. Immunology. 1997;92(3):388‐395. 9486113 10.1046/j.1365-2567.1997.00360.x PMC1363801

[50] Ouwehand K , Santegoets SJ , Bruynzeel DP , Scheper RJ , de Gruijl TD , Gibbs S . CXCL12 is essential for migration of activated Langerhans cells from epidermis to dermis. Eur J Immunol. 2008;38(11):3050‐3059. 18924211 10.1002/eji.200838384

[51] Kabashima K , Shiraishi N , Sugita K , et al. CXCL12‐CXCR4 engagement is required for migration of cutaneous dendritic cells. Am J Pathol. 2007;171(4):1249‐1257. 17823289 10.2353/ajpath.2007.070225 PMC1988874

[52] Wang Z , Wang W , Chai Q , Zhu M . Langerhans cells control lymphatic vessel function during inflammation via LIGHT‐LTbetaR signaling. J Immunol. 2019;202(10):2999‐3007. 30952816 10.4049/jimmunol.1801578

[53] Furio L , Briotet I , Journeaux A , Billard H , Peguet‐Navarro J . Human langerhans cells are more efficient than CD14(−)CD1c(+) dermal dendritic cells at priming naive CD4(+) T cells. J Invest Dermatol. 2010;130(5):1345‐1354. 20107482 10.1038/jid.2009.424

[54] Levin C , Bonduelle O , Nuttens C , et al. Critical role for skin‐derived migratory DCs and Langerhans cells in TFH and GC responses after intradermal immunization. J Invest Dermatol. 2017;137(9):1905‐1913. 28457909 10.1016/j.jid.2017.04.016

[55] Peiser M , Koeck J , Kirschning CJ , Wittig B , Wanner R . Human Langerhans cells selectively activated via Toll‐like receptor 2 agonists acquire migratory and CD4+T cell stimulatory capacity. J Leukoc Biol. 2008;83(5):1118‐1127. 18252867 10.1189/jlb.0807567

[56] Kumar H , Kawai T , Akira S . Toll‐like receptors and innate immunity. Biochem Biophys Res Commun. 2009;388(4):621‐625. 19686699 10.1016/j.bbrc.2009.08.062

[57] Flacher V , Bouschbacher M , Verronèse E , et al. Human Langerhans cells express a specific TLR profile and differentially respond to viruses and Gram‐positive bacteria. J Immunol. 2006;177(11):7959‐7967. 17114468 10.4049/jimmunol.177.11.7959

[58] Gramlich R , Aliahmadi E , Peiser M . In vitro induction of T helper 17 cells by synergistic activation of human monocyte‐derived langerhans cell‐like cells with bacterial agonists. Int J Mol Sci. 2019;20(6):1367. 10.3390/ijms20061367 PMC6471444 30893757

[59] Walsh KP , Mills KH . Dendritic cells and other innate determinants of T helper cell polarisation. Trends Immunol. 2013;34(11):521‐530. 23973621 10.1016/j.it.2013.07.006

[60] Ito T , Inaba M , Inaba K , et al. A CD1a+/CD11c+ subset of human blood dendritic cells is a direct precursor of Langerhans cells. J Immunol. 1999;163(3):1409‐1419. 10415041

[61] Reid CD , Fryer PR , Clifford C , Kirk A , Tikerpae J , Knight SC . Identification of hematopoietic progenitors of macrophages and dendritic Langerhans cells (DL‐CFU) in human bone marrow and peripheral blood. Blood. 1990;76(6):1139‐1149. 2400808

[62] Caux C , Massacrier C , Vanbervliet B , et al. CD34+ hematopoietic progenitors from human cord blood differentiate along two independent dendritic cell pathways in response to GM‐CSF+TNF alpha. Adv Exp Med Biol. 1997;417:21‐25. 9286332 10.1007/978-1-4757-9966-8_4

[63] Nguyen VA , Dubrac S , Forstner M , et al. CD34+ ‐derived Langerhans cell‐like cells are different from epidermal Langerhans cells in their response to thymic stromal lymphopoietin. J Cell Mol Med. 2011;15(9):1847‐1856. 21054781 10.1111/j.1582-4934.2010.01206.x PMC3918041

[64] Borkowski TA , Letterio JJ , Farr AG , Udey MC . A role for endogenous transforming growth factor beta 1 in Langerhans cell biology: the skin of transforming growth factor beta 1 null mice is devoid of epidermal Langerhans cells. J Exp Med. 1996;184(6):2417‐2422. 8976197 10.1084/jem.184.6.2417 PMC2196398

[65] Liu Y‐J , Soumelis V , Watanabe N , et al. TSLP: an epithelial cell cytokine that regulates T cell differentiation by conditioning dendritic cell maturation. Annu Rev Immunol. 2007;25:193‐219. 17129180 10.1146/annurev.immunol.25.022106.141718

[66] Ebner S , Nguyen VA , Forstner M , et al. Thymic stromal lymphopoietin converts human epidermal Langerhans cells into antigen‐presenting cells that induce proallergic T cells. J Allergy Clin Immunol. 2007;119(4):982‐990. 17320941 10.1016/j.jaci.2007.01.003

[67] Soumelis V , Reche PA , Kanzler H , et al. Human epithelial cells trigger dendritic cell mediated allergic inflammation by producing TSLP. Nat Immunol. 2002;3(7):673‐680. 12055625 10.1038/ni805

[68] Baker BS , Swain AF , Griffiths CE , Leonard JN , Fry L , Valdimarsson H . Epidermal T lymphocytes and dendritic cells in chronic plaque psoriasis: the effects of PUVA treatment. Clin Exp Immunol. 1985;61(3):526‐534. 3878241 PMC1577286

[69] Fujita H , Shemer A , Suárez‐Fariñas M , et al. Lesional dendritic cells in patients with chronic atopic dermatitis and psoriasis exhibit parallel ability to activate T‐cell subsets. J Allergy Clin Immunol. 2011;128(3):574‐582.e12. 21704361 10.1016/j.jaci.2011.05.016

[70] Komine M , Karakawa M , Takekoshi T , et al. Early inflammatory changes in the "perilesional skin" of psoriatic plaques: is there interaction between dendritic cells and keratinocytes? J Invest Dermatol. 2007;127(8):1915‐1922. 17446902 10.1038/sj.jid.5700799

[71] Bos JD , Hulsebosch HJ , Krieg SR , Bakker PM , Cormane RH . Immunocompetent cells in psoriasis. In situ immunophenotyping by monoclonal antibodies. Arch Dermatol Res. 1983;275(3):181‐189. 6604503 10.1007/BF00510050

[72] Lisi P . Investigation on Langerhans cells in pathological human epidermis. Acta Derm Venereol. 1973;53(6):425‐428. 4129586

[73] Gommans JM , van Hezik SJ , van Huystee BE . Flow cytometric quantification of T6‐positive cells in psoriatic epidermis after PUVA and methotrexate therapy. Br J Dermatol. 1987;116(5):661‐666. 3496110 10.1111/j.1365-2133.1987.tb05899.x

[74] Clarke LE , Helm KF , Hennessy J , Bruggeman RD , Clarke JT . Dermal dendritic cells in psoriasis, nummular dermatitis, and normal‐appearing skin. J Am Acad Dermatol. 2012;66(1):98‐105. 21669473 10.1016/j.jaad.2010.12.001

[75] Henseler T , Christophers E . Psoriasis of early and late onset: characterization of two types of psoriasis vulgaris. J Am Acad Dermatol. 1985;13(3):450‐456. 4056119 10.1016/s0190-9622(85)70188-0

[76] Shaw FL , Kimber I , Begum R , Cumberbatch M , Dearman RJ , Griffiths CE . No impairment of monocyte‐derived Langerhans cell phenotype or function in early‐onset psoriasis. Clin Exp Dermatol. 2012;37(1):40‐47. 21933242 10.1111/j.1365-2230.2011.04172.x PMC3298657

[77] Cumberbatch M , Singh M , Dearman RJ , Young HS , Kimber I , Griffiths CE . Impaired Langerhans cell migration in psoriasis. J Exp Med. 2006;203(4):953‐960. 16567387 10.1084/jem.20052367 PMC2118293

[78] Eaton LH , Chularojanamontri L , Ali FR , et al. Guttate psoriasis is associated with an intermediate phenotype of impaired Langerhans cell migration. Br J Dermatol. 2014;171(2):409‐411. 24628096 10.1111/bjd.12960

[79] Eaton LH , Dearman RJ , Kimber I , Griffiths CEM . Keratinocytes derived from late‐onset‐psoriasis skin do not impair Langerhans cell migration. Br J Dermatol. 2018;179(5):1208‐1209. 29923325 10.1111/bjd.16896

[80] Shaw FL , Cumberbatch M , Elise Kleyn C , et al. Langerhans cell mobilization distinguishes between early‐onset and late‐onset psoriasis. J Invest Dermatol. 2010;130(7):1940‐1942. 20237494 10.1038/jid.2010.57

[81] Miossec P , Korn T , Kuchroo VK . Interleukin‐17 and type 17 helper T cells. N Engl J Med. 2009;361(9):888‐898. 19710487 10.1056/NEJMra0707449

[82] Mavropoulos A , Rigopoulou EI , Liaskos C , Bogdanos DP , Sakkas LI . The role of p38 MAPK in the aetiopathogenesis of psoriasis and psoriatic arthritis. Clin Dev Immunol. 2013;2013:569751. 24151518 10.1155/2013/569751 PMC3787653

[83] Mihara K , Elliott GR , Boots AM , Nelissen RL . Inhibition of p38 kinase suppresses the development of psoriasis‐like lesions in a human skin transplant model of psoriasis. Br J Dermatol. 2012;167(2):455‐457. 22413906 10.1111/j.1365-2133.2012.10939.x

[84] Zheng T , Zhao W , Li H , et al. p38alpha signaling in Langerhans cells promotes the development of IL‐17‐producing T cells and psoriasiform skin inflammation. Sci Signal. 2018;11(521):eaao1685. 29535261 10.1126/scisignal.aao1685

[85] Sano S , Chan KS , DiGiovanni J . Impact of Stat3 activation upon skin biology: a dichotomy of its role between homeostasis and diseases. J Dermatol Sci. 2008;50(1):1‐14. 17601706 10.1016/j.jdermsci.2007.05.016

[86] Nakajima K , Kataoka S , Sato K , et al. Stat3 activation in epidermal keratinocytes induces Langerhans cell activation to form an essential circuit for psoriasis via IL‐23 production. J Dermatol Sci. 2019;93(2):82‐91. 30514663 10.1016/j.jdermsci.2018.11.007

[87] Lande R , Chamilos G , Ganguly D , et al. Cationic antimicrobial peptides in psoriatic skin cooperate to break innate tolerance to self‐DNA. Eur J Immunol. 2015;45(1):203‐213. 25332209 10.1002/eji.201344277

[88] Sweeney CM , Russell SE , Malara A , et al. Human ss‐defensin 3 and its mouse ortholog murine ss‐defensin 14 activate Langerhans cells and exacerbate psoriasis‐like skin inflammation in mice. J Invest Dermatol. 2016;136(3):723‐727. 27015459 10.1016/j.jid.2015.12.011

[89] Wolk K , Witte E , Wallace E , et al. IL‐22 regulates the expression of genes responsible for antimicrobial defense, cellular differentiation, and mobility in keratinocytes: a potential role in psoriasis. Eur J Immunol. 2006;36(5):1309‐1323. 16619290 10.1002/eji.200535503

[90] Duhen T , Geiger R , Jarrossay D , Lanzavecchia A , Sallusto F . Production of interleukin 22 but not interleukin 17 by a subset of human skin‐homing memory T cells. Nat Immunol. 2009;10(8):857‐863. 19578369 10.1038/ni.1767

[91] Fujita H , Nograles KE , Kikuchi T , Gonzalez J , Carucci JA , Krueger JG . Human Langerhans cells induce distinct IL‐22‐producing CD4+ T cells lacking IL‐17 production. Proc Natl Acad Sci USA. 2009;106(51):21795‐21800. 19996179 10.1073/pnas.0911472106 PMC2799849

[92] Oesch F , Fabian E , Landsiedel R . Xenobiotica‐metabolizing enzymes in the skin of rat, mouse, pig, guinea pig, man, and in human skin models. Arch Toxicol. 2018;92(8):2411‐2456. 29916051 10.1007/s00204-018-2232-x PMC6063329

[93] Kim TG , Kim SH , Lee MG . The origin of skin dendritic cell network and its role in psoriasis. Int J Mol Sci. 2018;19(1):42. 10.3390/ijms19010042 PMC5795992 29295520

[94] Bennett CL , van Rijn E , Jung S , et al. Inducible ablation of mouse Langerhans cells diminishes but fails to abrogate contact hypersensitivity. J Cell Biol. 2005;169(4):569‐576. 15897263 10.1083/jcb.200501071 PMC2171694

[95] Bobr A , Olvera‐Gomez I , Igyarto BZ , Haley KM , Hogquist KA , Kaplan DH . Acute ablation of Langerhans cells enhances skin immune responses. J Immunol. 2010;185(8):4724‐4728. 20855870 10.4049/jimmunol.1001802 PMC3050031

[96] van der Fits L , Mourits S , Voerman JSA , et al. Imiquimod‐induced psoriasis‐like skin inflammation in mice is mediated via the IL‐23/IL‐17 axis. J Immunol. 2009;182(9):5836‐5845. 19380832 10.4049/jimmunol.0802999

[97] Zheng Y , Danilenko DM , Valdez P , et al. Interleukin‐22, a T(H)17 cytokine, mediates IL‐23‐induced dermal inflammation and acanthosis. Nature. 2007;445(7128):648‐651. 17187052 10.1038/nature05505

[98] Zenz R , Eferl R , Kenner L , et al. Psoriasis‐like skin disease and arthritis caused by inducible epidermal deletion of Jun proteins. Nature. 2005;437(7057):369‐375. 16163348 10.1038/nature03963

[99] Xiao C , Zhu Z , Sun S , et al. Activation of Langerhans cells promotes the inflammation in imiquimod‐induced psoriasis‐like dermatitis. J Dermatol Sci. 2017;85(3):170‐177. 27964879 10.1016/j.jdermsci.2016.12.003

[100] Lowes MA , Suarez‐Farinas M , Krueger JG . Immunology of psoriasis. Annu Rev Immunol. 2014;32:227‐255. 24655295 10.1146/annurev-immunol-032713-120225 PMC4229247

[101] Singh TP , Lee CH , Farber JM . Chemokine receptors in psoriasis. Expert Opin Ther Targets. 2013;17(12):1405‐1422. 24070343 10.1517/14728222.2013.838220

[102] Suarez‐Farinas M , Arbeit R , Jiang W , Ortenzio FS , Sullivan T , Krueger JG . Suppression of molecular inflammatory pathways by Toll‐like receptor 7, 8, and 9 antagonists in a model of IL‐23‐induced skin inflammation. PLoS ONE. 2013;8(12):e84634. 24386404 10.1371/journal.pone.0084634 PMC3874038

[103] Roller A , Perino A , Dapavo P , et al. Blockade of phosphatidylinositol 3‐kinase PI3Kdelta or PI3Kgamma reduces IL‐17 and ameliorates imiquimod‐induced psoriasis‐like dermatitis. J Immunol. 2012;189(9):4612‐4620. 23024273 10.4049/jimmunol.1103173

[104] Yoshiki R , Kabashima K , Honda T , et al. IL‐23 from Langerhans cells is required for the development of imiquimod‐induced psoriasis‐like dermatitis by induction of IL‐17A‐producing gammadelta T cells. J Invest Dermatol. 2014;134(7):1912‐1921. 24569709 10.1038/jid.2014.98

[105] Cua DJ , Tato CM . Innate IL‐17‐producing cells: the sentinels of the immune system. Nat Rev Immunol. 2010;10(7):479‐489. 20559326 10.1038/nri2800

[106] Kim JH , Hu YU , Yongqing T , et al. CD1a on Langerhans cells controls inflammatory skin disease. Nat Immunol. 2016;17(10):1159‐1166. 27548435 10.1038/ni.3523 PMC5791155

[107] Flutter B , Nestle FO . TLRs to cytokines: mechanistic insights from the imiquimod mouse model of psoriasis. Eur J Immunol. 2013;43(12):3138‐3146. 24254490 10.1002/eji.201343801

[108] Terhorst D , Chelbi R , Wohn C , et al. Dynamics and transcriptomics of skin dendritic cells and macrophages in an imiquimod‐induced, biphasic mouse model of psoriasis. J Immunol. 2015;195(10):4953‐4961. 26466959 10.4049/jimmunol.1500551

[109] Glitzner E , Korosec A , Brunner PM , et al. Specific roles for dendritic cell subsets during initiation and progression of psoriasis. EMBO Mol Med. 2014;6(10):1312‐1327. 25216727 10.15252/emmm.201404114 PMC4287934

[110] Kanda N , Watanabe S . IL‐12, IL‐23, and IL‐27 enhance human beta‐defensin‐2 production in human keratinocytes. Eur J Immunol. 2008;38(5):1287‐1296. 18389480 10.1002/eji.200738051

[111] Nakajima S , Igyártó BZ , Honda T , et al. Langerhans cells are critical in epicutaneous sensitization with protein antigen via thymic stromal lymphopoietin receptor signaling. J Allergy Clin Immunol. 2012;129(4):1048‐1055.e1046. 22385635 10.1016/j.jaci.2012.01.063 PMC4600611

[112] Stene MA , Babajanians M , Bhuta S , Cochran AJ . Quantitative alterations in cutaneous Langerhans cells during the evolution of malignant melanoma of the skin. J Invest Dermatol. 1988;91(2):125‐128. 3260930 10.1111/1523-1747.ep12464142

